# Beyond bactericidal: targeting plasmid-mediated antibiotic resistance with natural product-based plasmid curing agents

**DOI:** 10.3389/fmicb.2026.1802582

**Published:** 2026-04-23

**Authors:** Jia Wang, Qun Liang

**Affiliations:** 1 Heilongjiang University of Chinese Medicine, Harbin, China; 2 The First Affiliated Hospital of Heilongjiang University of Chinese Medicine, Harbin, China

**Keywords:** antibiotic resistance, horizontal gene transfer, natural products, plasmid curing, reversal of resistance

## Abstract

Antimicrobial resistance (AMR) has evolved into a severe global public health crisis, with plasmid-mediated horizontal gene transfer (HGT) serving as a core driver for the rapid dissemination of multidrug resistance (MDR). Traditional “bactericidal” antibiotic strategies impose strong selective pressure, failing to eradicate the root cause of resistance while accelerating the enrichment of resistant clones. “Plasmid curing”—a strategy that specifically eliminates resistance plasmids to restore antibiotic susceptibility—has emerged as a promising paradigm shift. While early synthetic curing agents suffered from severe cytotoxicity, natural products (e.g., alkaloids, quinones, terpenoids) exhibit unique potential owing to their structural diversity and multi-target profiles. This review systematically elucidates the molecular mechanisms by which natural products achieve plasmid eradication, including the disruption of Rep-ori replication initiation, interference with ParA-ParB partitioning dynamics, and the blockade of conjugation via type IV secretion system (T4SS) and quorum sensing (QS) inhibition. Crucially, we critically evaluate the methodological workflows—from high-throughput screening to absolute quantitative PCR—necessary to strictly differentiate true *in vivo* plasmid curing from mere selective bactericidal artifacts. Furthermore, we address current translational bottlenecks, particularly the “therapeutic window paradox,” and highlight how integrating advanced nanotechnology, artificial intelligence (AI)-guided drug discovery, and CRISPR-Cas9 synergies will propel the field forward. By shifting the therapeutic paradigm from violent “bacterial killing” to ecologically intelligent “genetic disarmament,” natural plasmid-curing agents offer a vital, adjunctive solution for safeguarding the lifespan of legacy antibiotics.

## 1 Introduction

The discovery of antibiotics stands as one of the most decisive achievements in 20th-century medicine. However, the ensuing “golden era” of antibiotic therapy is rapidly fading, giving way to the advent of a “post-antibiotic era.” A recent comprehensive report in The Lancet revealed that in 2019, approximately 1.27 million deaths were directly attributable to AMR, while 4.95 million deaths were indirectly associated with it. It is projected that between 2025 and 2050, deaths directly caused by bacterial drug resistance will exceed 39 million, with indirectly related deaths potentially reaching as high as 169 million (Antimicrobial Resistance Collaborators, 2022). The World Health Organization (WHO) has identified AMR as one of the top ten global public health threats facing humanity (Antimicrobial Resistance Collaborators, 2022). A key, yet often underestimated, driver of this crisis is HGT, particularly that mediated by mobile genetic elements. Among these, plasmids—extrachromosomal DNA molecules capable of autonomous replication—serve as the primary vectors for disseminating resistance genes (R-genes) and are central drivers of this process. For instance, genes encoding extended-spectrum *β*-lactamases (ESBLs, such as blaCTX-M, and blaNDM-1, and blaKPC) enable susceptible bacteria to acquire a “ready-made” multidrug-resistant phenotype without any chromosomal mutations (Carattoli, 2013). Consequently, plasmids are not merely mobile carriers of resistance genes but also critical weapons that allow resistant bacteria to persistently “evade” the effects of antimicrobial agents on a global scale (Partridge et al., 2018; San Millan, 2018).

Over the past century, therapeutic strategies have relied almost exclusively on bactericidal or bacteriostatic agents. For instance, *β*-lactam antibiotics inhibit cell wall synthesis, quinolones interfere with DNA replication, and aminoglycosides suppress protein translation—all of which ultimately lead to bacterial death. However, this bactericidal-centered treatment approach exhibits limitations in addressing bacterial drug resistance: even when susceptible bacteria are rapidly eliminated, the resistance plasmids they carry can still spread within the microbial community through horizontal gene transfer, continuously disseminating resistance genes. Moreover, the selective pressure exerted by antibiotics has a dual effect: while clearing susceptible strains, it also provides an ecological advantage to strains with resistance mutations, promoting the colonization and transmission of resistant clones. Consequently, research focus has gradually shifted toward “plasmid curing”—a strategy aimed at eliminating resistance plasmids (R-plasmids) from bacterial hosts rather than directly killing the bacteria (Buckner et al., 2018). By removing the genetic determinants of resistance, bacteria are effectively reverted to a susceptible phenotype, rendering them vulnerable again to traditional narrow-spectrum antibiotics.

Early synthetic plasmid-curing agents, such as acridine orange and ethidium bromide, were identified as potent plasmid eliminators. However, due to their mechanism of action—intercalating into DNA double strands to interfere with plasmid replication—they lack selectivity and exhibit significant cytotoxicity and potential genotoxicity, thereby limiting their clinical application. In this context, plant-derived natural products and phytochemicals, characterized by their multi-component and multi-target properties, offer potential for developing novel, low-toxicity plasmid-curing strategies. Existing studies have shown that various phytochemical extracts and diverse natural compounds—such as berberine derived from Coptis chinensis (Boberek et al., 2010; Saha et al., 2026) and baicalin from Scutellaria baicalensis (Chen et al., 2025)—not only possess antibacterial activity but can also promote the loss of resistance plasmids in bacteria without exerting strong bactericidal selective pressure by affecting plasmid stability, replication, or conjugation transfer processes.

Research on plasmid elimination based on globally investigated natural products offers a novel approach distinct from conventional bactericidal strategies to address bacterial drug resistance. This review aims to provide a comprehensive analysis of natural products with plasmid-eliminating activity. We systematically categorize these agents—including alkaloids, flavonoids, quinones, and terpenoids—delve into their molecular mechanisms of action—ranging from inhibition of plasmid replication to blockade of conjugation transfer—and discuss their potential as clinical adjuvants against multidrug-resistant superbugs.

## 2 Mechanisms of plasmid maintenance and transfer: potential targets

Understanding the molecular mechanisms underlying plasmid maintenance and horizontal transfer within bacterial populations is a prerequisite for developing specific “curing agents.” Unlike broad-spectrum antibiotics, which non-specifically disrupt essential bacterial life processes—such as cell wall synthesis, ribosomal protein synthesis, nucleic acid replication, and folate metabolism—plasmid curing strategies aim to precisely interfere with plasmid-specific survival pathways: replication, partitioning, addiction systems, and conjugative transfer. These plasmid-specific molecular machines constitute ideal drug targets, providing a theoretical foundation for the development of plasmid curing agents (Nordström and Austin, 1989).

### 2.1 Plasmid replication

**Figure 1:**
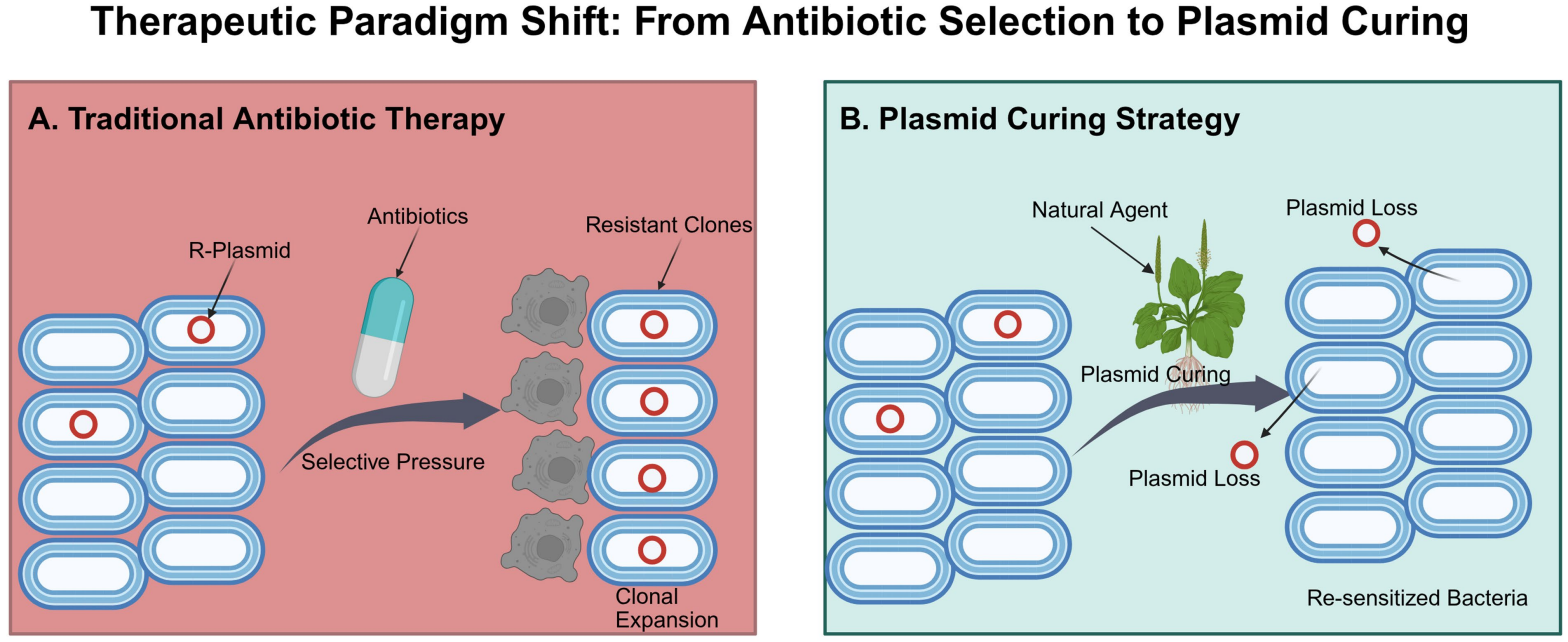
Therapeutic paradigm shift: from antibiotic selection to plasmid curing (created with BioRender.com). **(A)** Traditional antibiotic therapy exerts strong selective pressure, eliminating susceptible bacteria while driving the clonal expansion and dominance of resistant populations. **(B)** The plasmid curing strategy utilizes natural agents to specifically induce resistance plasmid loss without bactericidal effects, effectively re-sensitizing the bacterial population to conventional antibiotics.

Plasmid replication is a fundamental biological process essential for maintaining the genetic stability of bacterial plasmids and facilitating their dissemination within host populations. Unlike chromosomal DNA, which relies on the host chromosome’s replication initiation system, plasmid replication is predominantly autonomous. This autonomy is achieved through the recognition of the plasmid-borne origin of replication (ori) by a specific replication initiation protein (Rep protein) encoded by the plasmid itself. This independence enables plasmids to replicate stably in diverse hosts and serves as a critical vehicle for horizontal gene transfer, particularly for antibiotic resistance and virulence genes. Plasmid replication primarily occurs via two distinct mechanisms: rolling-circle replication and theta replication. These mechanisms exhibit significant differences in their molecular pathways, regulatory strategies, and dependence on host factors. These differences also present potential targets for the selective intervention of plasmid replication (Figure 1).

#### 2.1.1 Rolling circle replication

Rolling circle replication (RCR) is commonly observed in small plasmids of many Gram-positive bacteria (e.g., the pT181 family plasmids in *Staphylococcus aureus*). The process initiates when the plasmid-encoded Rep protein generates a specific single-strand nick on the positive strand at the double-strand origin (dso). Subsequently, the Rep protein covalently attaches to the 5′ end generated by the nick and, utilizing the host-provided DNA polymerase III, uses the intact negative strand as a template to extend from the 3′-OH end at the nick site, progressively displacing the original positive strand. The displaced positive strand is then synthesized into a complementary strand by the host polymerase I, ultimately forming a double-stranded replication intermediate. Replication often produces linear concatemers, which require cleavage by a specific mechanism to generate monomeric circular plasmids (Khan, 2005).

**Figure 2:**
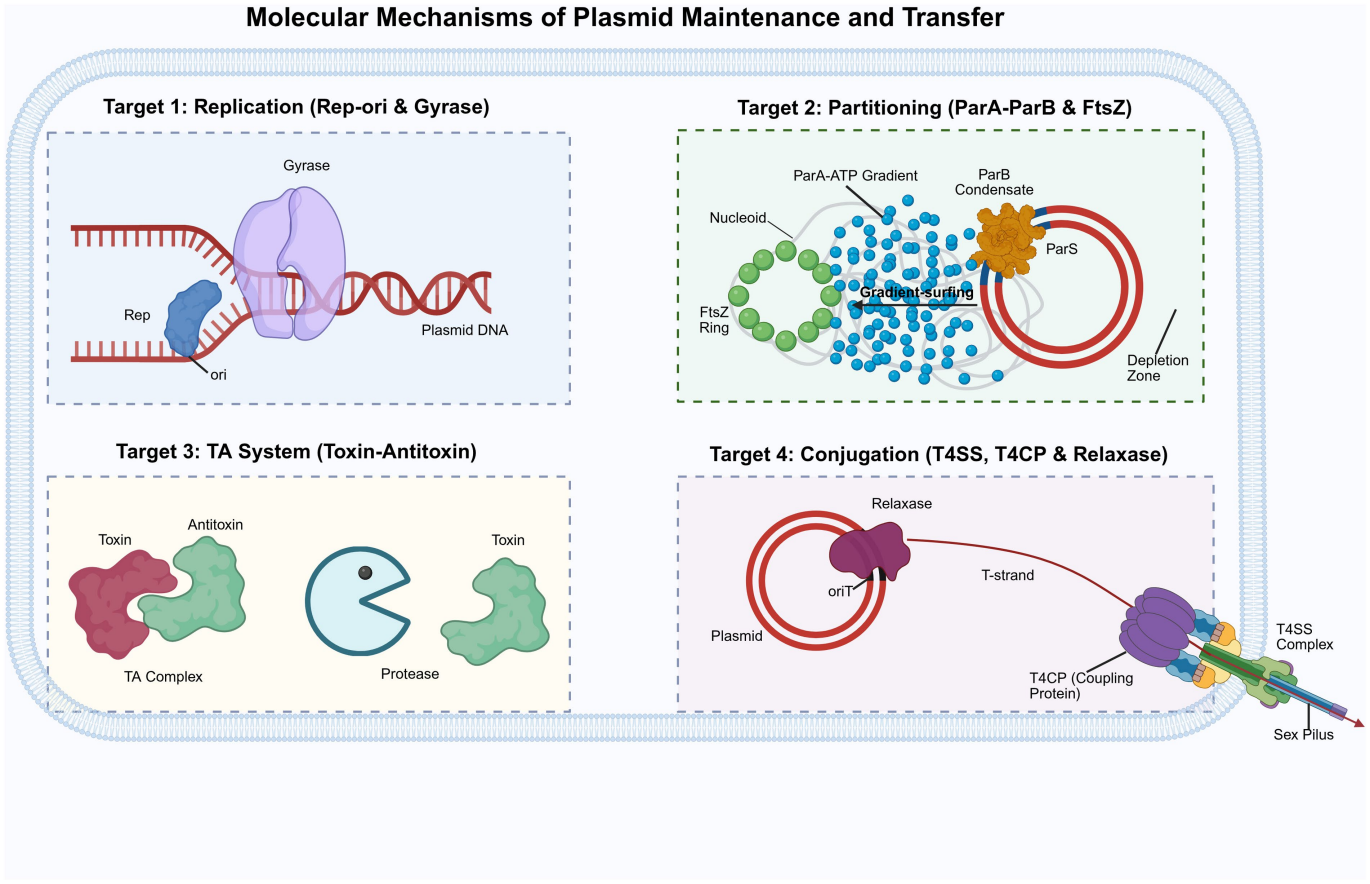
Molecular mechanisms of plasmid maintenance and transfer (created with BioRender.com). Overview of four key plasmid-specific vulnerabilities targeted by natural curing agents. (1) Plasmid replication: Disruption of the Rep-ori initiation complex and associated host factors (e.g., DNA gyrase). (2) Partitioning system: Interference with the ParA/ParB segregation machinery. The schematic highlights ParB condensation at the *parS* site and the ParA-ATP gradient-driven dynamics (gradient-surfing) across the host nucleoid, which leaves a characteristic depletion zone during plasmid movement. (3) Toxin-antitoxin (TA) system: Targeting plasmid addiction modules (toxin-antitoxin complexes and proteases) to prevent post-segregational killing. (4) Conjugation: Blockade of the Type IV Secretion System (T4SS) channel, relaxase activity, or the essential T4CP (coupling protein) motor to halt the horizontal transfer of the T-strand.

Although the elongation phase of RCR is highly dependent on the host’s fundamental replication machinery (e.g., polymerases, helicases), the initiation step is entirely autonomous and plasmid-specific. Crucially, the Rep proteins driving RCR are typically HUH endonucleases, which share no structural homologues with the host bacteria’s primary replication initiator, DnaA. Furthermore, the plasmid’s dso sequence lacks any meaningful sequence similarity to the bacterial chromosomal origin (oriC). Because of this profound evolutionary and structural divergence, natural curing agents that specifically inhibit the unique endonuclease activity of the RCR Rep protein or block its binding to the dso can selectively arrest plasmid replication. This precise intervention prevents plasmid duplication without interfering with the host’s native chromosomal replication cycle, thereby strictly avoiding host cytotoxicity (Figure 2).

#### 2.1.2 Theta replication

Theta replication is the predominant mode of replication for large plasmids in most Gram-negative bacteria, such as common conjugative plasmids like the F plasmid and the R1 plasmid. Replication initiates when the Rep protein recognizes and binds to the replication origin (oriV), forming a replication bubble. Subsequently, bidirectional replication forks extend until they meet and complete replication. Based on the degree of dependence on the Rep protein, Theta replication can be classified into subtypes such as those reliant on plasmid-encoded Rep proteins (e.g., IncF and IncP plasmids) and those primarily dependent on host RNA polymerase for primer synthesis (e.g., ColE1-type plasmids) (Del Solar et al., 1998).

For Rep-dependent plasmids, autonomous replication is achieved by encoding specific Rep proteins (e.g., RepE, RepA) and developing sophisticated copy number control networks (Chattoraj, 2000). Structural and functional studies reveal that the sequence similarity between these plasmid Rep proteins and the host chromosomal initiator DnaA is remarkably low. Plasmid Rep proteins typically utilize distinct winged-helix domains to specifically bind to repetitive sequences (iterons) within oriV, which in turn share no significant sequence homology with the DnaA-boxes found in the host oriC (Wegrzyn et al., 2014; Wolański et al., 2015).

**Figure 3:**
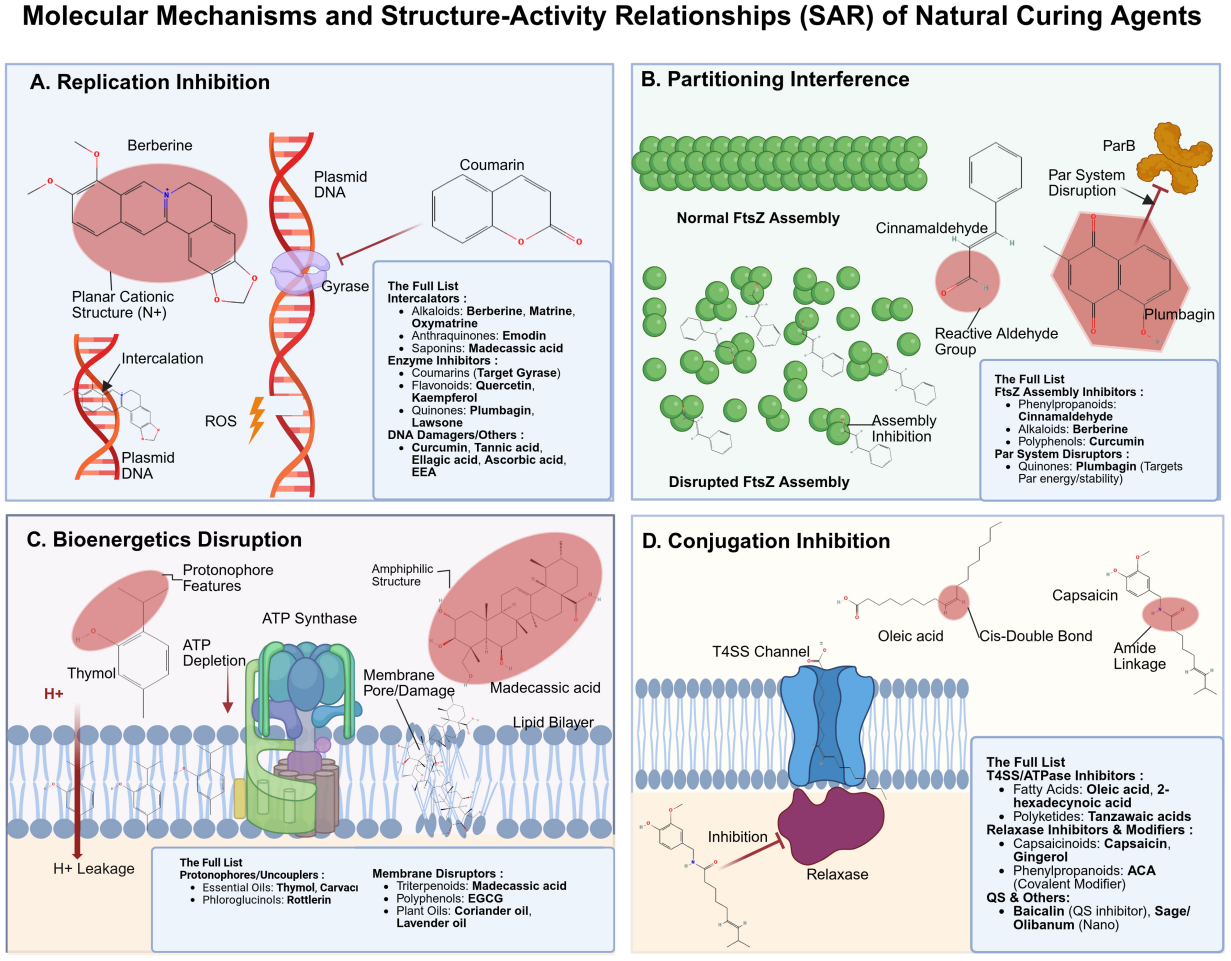
Molecular mechanisms and structure–activity relationships (SAR) of natural curing agents (Created with BioRender.com and ChemDraw Professional). Visualization of natural product interactions with specific biological targets. **(A)** Replication inhibition: Agents such as berberine (DNA intercalation) and coumarins (gyrase inhibition) block replication initiation. **(B)** Partitioning interference: Compounds like cinnamaldehyde and plumbagin disrupt FtsZ Z-ring assembly or Par system stability. **(C)** Bioenergetics disruption: Lipophilic molecules (e.g., thymol, madecassic acid) act as protonophores or disrupt membrane integrity, dissipating the proton motive force (PMF) and causing ATP depletion. **(D)** Conjugation inhibition: Unsaturated fatty acids, ACA, and flavonoids (e.g., baicalin) halt horizontal transfer by blocking the T4SS channel, covalently modifying key conjugation proteins, or quenching quorum sensing (QS) signals. T4SS, Type IV secretion system; QS, Quorum sensing; PMF, Proton motive force.

This extreme structural divergence addresses a fundamental paradox in plasmid curing: since Theta replication deeply hijacks the host’s fundamental replication machinery (DNA polymerases, helicases, gyrases) for chain elongation, how can curing agents avoid killing the host? The answer lies in precisely targeting the initiation phase rather than the elongation phase. By utilizing natural agents that act as competitive inhibitors of the unique Rep-oriV interaction or as allosteric modulators of Rep dimerization interfaces, researchers can successfully abort plasmid replication at the very first step. This strategy leaves the host’s DnaA-oriC interaction and subsequent replication machinery completely unperturbed (Khan, 2005; Lu et al., 1998). Furthermore, for conjugative plasmids, this Rep-mediated replication is often functionally coupled with the conjugation transfer mechanism, making the Rep-oriV complex a critical and highly selective target for halting the widespread dissemination of antibiotic resistance genes without threatening bacterial survival (Figure 3).

#### 2.1.3 Target relevance

The plasmid replication mechanism shares certain fundamental elements with host chromosomal replication while possessing unique critical nodes. This provides a solid biological foundation for developing “plasmid-curing agents” capable of selectively interfering with plasmid replication without lethally affecting the survival of the host bacterium.

##### 2.1.3.1 Specificity of structure and sequence

Plasmid-specific Rep proteins and bacterial chromosomal replication initiation proteins (e.g., DnaA) lack detectable primary sequence homology. Because they have evolved from entirely divergent lineages, standard sequence alignment algorithms cannot compute a meaningful percentage identity. Structural and functional studies reveal that while DnaA relies on a highly conserved DNA-binding domain to recognize the specific chromosomal origin (oriC), many plasmid Rep proteins (e.g., RepA, RepF) utilize distinct Winged-Helix domains to bind their highly unique plasmid replication origins (oriV) (Wegrzyn et al., 2014; Wolański et al., 2015). Astonishingly, structural phylogenetics indicate that many plasmid Rep proteins share greater structural homology with eukaryotic and archaeal replication initiators (e.g., Orc4p/Cdc6p) than with bacterial DnaA (Giraldo and Diaz-Orejas, 2001). Furthermore, at the nucleotide level, functional studies demonstrate that oriV and oriC sequences are fundamentally non-interchangeable; replacing key oriV motifs with corresponding oriC sequences completely inactivates the plasmid replication origin (Kowalczyk et al., 2005). This profound evolutionary and structural divergence at the molecular level ensures that interventions specifically targeting the Rep-oriV interaction do not inadvertently disrupt the DnaA-oriC chromosomal replication process, thereby achieving high selectivity and minimizing host cytotoxicity (Lu et al., 1998).

##### 2.1.3.2 Unique dependence on topology and selectivity limits

Although both plasmids and chromosomes are regulated by topoisomerases, plasmids—being significantly smaller and often possessing different supercoiling densities—are highly sensitive to topological alterations. The initiation of plasmid replication strictly requires specific topological conformations. However, as topology-altering agents (e.g., planar intercalators) often lack absolute target specificity, their intercalation can stabilize topoisomerase II-DNA cleavage complexes within the host chromosome, leading to off-target DNA double-strand breaks and host cytotoxicity (Li et al., 2010; Müller et al., 1996). Therefore, the therapeutic window of these agents relies entirely on exploiting the quantitative differences in supercoiling dependence between plasmids and host chromosomes without triggering severe host genotoxicity.

##### 2.1.3.3 Non-essentiality of the host-plasmid protein–protein interaction (PPI) interfaces

To utilize the host’s fundamental replication machinery, plasmids have evolved specific PPI interfaces that act as critical recruitment nodes. For instance, plasmid-encoded Rep proteins typically utilize distinct hydrophobic interaction interfaces to directly recruit and load the host’s DnaB helicase or DnaG primase onto the plasmid origin (oriV) (Del Solar et al., 1998). Crucially, these specific Rep-host interfaces are entirely non-essential for the host’s own survival, as the host relies on its native DnaA protein to recruit DnaB at the chromosomal origin (oriC). Therefore, disrupting these specific Rep-helicase interaction points can selectively “cut off” the plasmid’s replication resource supply without compromising the host’s core chromosomal DNA synthesis.

#### 2.1.4 Potential intervention strategies

Based on the specificity and vulnerability of the aforementioned targets, drug design can focus on disrupting the assembly of the replication initiation complex. Specific strategies are proposed from the perspective of “how to do it”:

*Competitive inhibition*: Nucleic acid analogs or small molecule ligands that mimic the structure of the ori sequence can be designed to serve as “molecular decoys.” These agents competitively bind to the DNA-binding domain of the Rep protein, thereby preventing its localization. However, a critical review of current literature reveals that true direct competitive inhibitors of the Rep-DNA interface remain a major unexplored gap. Most discoveries come from phenotypic high-throughput screening. For instance, the nucleoside analog CGS 15943 exhibits complete suppression of plasmid replication by inhibiting RepE function, though its exact competitive binding mode requires structural validation (Zulauf and Kirby, 2020). Similarly, natural phenolic compounds (e.g., quercetin and bharangin) have been shown to specifically cure iteron-containing, Rep-dependent plasmids while leaving ColE1-type plasmids unaffected, suggesting highly specific interference with the Rep initiation complex (Lakshmi et al., 1989).

*Allosteric modulation*: This approach involves identifying small molecules that bind to non-active sites of the Rep protein, inducing conformational changes that impair its ability to recognize the ori or recruit host helicases. This strategy is generally less prone to the development of drug resistance compared to direct competition for the active site. A well-characterized example involves natural flavones (such as myricetin and dimyricetin), which act as potent non-competitive inhibitors of the hexameric replicative helicase RepA. Rather than competing for the ATP-binding pocket, these phytochemicals bind to an allosteric site, effectively impairing helicase unwinding activity (Xu et al., 2001). Furthermore, certain natural products can indirectly achieve similar outcomes by selectively depleting Rep proteins; for example, the natural aminoglycoside kasugamycin specifically inhibits the translation of RepE mRNA, leading to the rapid eviction of multidrug-resistance plasmids (Zulauf and Kirby, 2020).

*Topological interference*: This strategy utilizes planar-structured molecules (e.g., classic intercalators like ethidium bromide, or natural anthraquinones like emodin and plumbagin) that intercalate between DNA base pairs, altering the local topology of the ori region. The resulting steric hindrance and topological changes physically blocks the binding of the Rep protein (Spengler et al., 2006). Regarding specificity, it is crucial to acknowledge that these planar molecules generally lack absolute selectivity for plasmid DNA over host chromosomal DNA. Their apparent “plasmid-specific” effect at sub-inhibitory concentrations relies on the fact that plasmids—due to their smaller size and distinct supercoiling densities—are often exquisitely more sensitive to topological alterations for replication initiation than the host chromosome. However, at higher concentrations, these intercalators inevitably stabilize topoisomerase II-DNA cleavage complexes within the host chromosome, leading to off-target DNA double-strand breaks and significant host cytotoxicity (Li et al., 2010; Müller et al., 1996). Therefore, developing non-intercalating strategies remains a priority.

### 2.2 Partitioning systems (Par systems)

A plasmid partitioning system is a molecular apparatus that ensures the accurate and active segregation of plasmids into both daughter cells during bacterial cell division. For low-copy-number plasmids, genetic stability cannot be guaranteed by random diffusion alone. Therefore, Par systems are crucial for preventing plasmid loss due to random segregation.

#### 2.2.1 Mechanism: the ParA-ParB-parS system and gradient-surfing dynamics

A canonical Par system comprises three core elements: a cis-acting centromere-like sequence (the parS site) and two trans-acting proteins—an ATPase (ParA) and a CTPase/DNA-binding protein (ParB). The partitioning process initiates with ParB condensation. Recent studies reveal that ParB functions as a CTP-dependent molecular switch. Upon CTP binding, ParB dimers nucleate at the parS site and undergo phase separation to form a liquid-like condensate (Delimi et al., 2025; Ti Ma et al., 2023; Zhao Y. et al., 2024). This CTP hydrolysis cycle triggers transient 1D sliding and 3D protein-DNA bridging, continuously condensing the flanking DNA into a robust, massive nucleoprotein partition complex (Connolley et al., 2023; Osorio-Valeriano et al., 2021; Osorio-Valeriano et al., 2019; Szymczak et al., 2025).

Following ParB condensation, plasmid movement is driven by a dynamic ParA concentration gradient. ParA-ATP dimers bind non-specifically to the host nucleoid, forming a dense, left-handed helical filament “carpet”(Chodha et al., 2023; Parker et al., 2021; Surovtsev et al., 2016). The large ParB-parS partition complex dynamically interacts with this nucleoid-bound ParA, highly stimulating its ATPase activity. This localized ATP hydrolysis causes ParA to dissociate from the DNA, generating a nucleoid-associated ParA depletion zone immediately behind the moving plasmid (Surovtsev et al., 2016). As the plasmid continuously diffuses up this concentration gradient toward regions of higher ParA-ATP density—a mechanism known as the “diffusion-ratchet” or “gradient-surfing” model—it is directionally transported across the nucleoid, ensuring accurate spatial segregation into daughter cells (Chodha et al., 2023; Surovtsev et al., 2016).

#### 2.2.2 Target relevance

The Par system, a plasmid-specific maintenance machinery, exhibits extremely low homology with host chromosomal segregation mechanisms. This profound structural and evolutionary divergence constitutes its core advantage as a highly selective drug target.

##### 2.2.2.1 System independence and the resolution of resistance

Bacterial chromosomal segregation primarily relies on structural maintenance of chromosomes (SMC) complexes, such as the MukBEF system in key pathogens like *Escherichia coli*. In stark contrast, plasmids rely on the highly distinct ParABS system. Because sequence homology between plasmid Par proteins and host MukBEF/SMC components is virtually non-existent (Mäkelä and Sherratt, 2020), specific Par inhibitors selectively induce plasmid mis-segregation without interfering with essential host cell division.

##### 2.2.2.2 This dynamic perfectly addresses a critical paradox

If bacteria survive Par inhibition, why does resistance not persist? The answer lies in asymmetric inheritance. When a plasmid fails to partition actively during cell division, it is inevitably lost in one of the resulting daughter cells. While these plasmid-free daughter cells are perfectly viable, they have been completely stripped of their plasmid-encoded resistance determinants. Consequently, they are restored to a fully susceptible state. When such curing agents are administered in combination with conventional antibiotics, this newly resensitized population is rapidly and safely eradicated (Buckner et al., 2018; Kamruzzaman et al., 2017). This aligns perfectly with the strategic requirement of “plasmid curing rather than bactericidal activity.”

##### 2.2.2.3 Component specificity for narrow-spectrum targeting

The interaction between the ParB protein and the parS centromere-like sequence is highly specific. Because parS sequences vary significantly among different plasmid incompatibility (Inc) groups, this specificity provides a robust structural basis for developing narrow-spectrum curing agents. Such agents could be tailored to precisely eradicate specific classes of multidrug-resistant plasmids from a bacterial population without disrupting the broader, beneficial microbiome.

#### 2.2.3 Potential intervention strategies

The “plasmid-curing” strategy targeting the Par system aims to paralyze its motor system or positioning anchor points:

*Inhibiting the motor*: Screening for small-molecule inhibitors that target the ATP-binding pocket of ParA to block ATP hydrolysis or inhibit the polymerization-depolymerization dynamics of ParA, thereby depriving it of the mechanical force required for “dragging” plasmids (Million-Weaver and Camps, 2014).

*Disrupting the positioning complex*: Utilizing peptidomimetics or small molecules to interfere with the specific binding between ParB and the parS sites, or to disrupt the anchoring of the Par system to the host cytoskeleton (e.g., FtsZ/MreB). This would cause plasmids to lose spatial positioning signals and drift randomly.

### 2.3 Post-segregational killing

Many MDR plasmids carry toxin-antitoxin (TA) systems (e.g., hok/sok, ccdB/ccdA), also known as plasmid addiction systems. This system represents another sophisticated strategy employed by plasmids to ensure their stable maintenance within a bacterial host population (Chan et al., 2015; Harms et al., 2018).

#### 2.3.1 Mechanism: toxin-antitoxin systems

##### 2.3.1.1 The system typically consists of two closely linked genes

One encoding a stable toxin protein and the other encoding an unstable antitoxin. When the plasmid is present, the antitoxin is constitutively expressed and neutralizes the toxin’s activity. However, if the plasmid is lost during cell division, the unstable antitoxin is rapidly degraded by host proteases, while the stable toxin persists. The toxin then exerts its toxic effect, killing or inhibiting the growth of progeny cells that have lost the plasmid. This process “penalizes” plasmid-free cells at the population level, thereby maintaining a high plasmid carriage rate (Page and Peti, 2016; Yamaguchi et al., 2011).

##### 2.3.1.2 Common types of TA systems include

Type I (where the antitoxin is an antisense RNA that inhibits toxin mRNA translation), Type II (where the antitoxin is a protein that directly binds and inhibits the toxin protein), and Type III (where the antitoxin RNA directly binds and inhibits the toxin protein). Many clinically significant resistance plasmids (e.g., IncF, IncA/C types) carry multiple TA systems, which enhances their genetic stability.

#### 2.3.2 Target relevance

Although the primary function of the TA system is plasmid maintenance, its unique mechanism also provides a rationale for “subverting” this system to achieve plasmid elimination. Its validity as a drug target lies in the uniqueness and vulnerability of its mechanism.

##### 2.3.2.1 Uniqueness of the “Addiction” mechanism

This “life-or-death binding” mechanism, based on the differential protein stability between the unstable antitoxin and the stable toxin, is not commonly found in the maintenance of essential genes within the host genome. It represents a burden imposed on the host by the plasmid.

##### 2.3.2.2 Intrinsic instability

The function of the TA system entirely depends on the continuous supply and rapid degradation of the antitoxin. This delicate balance is highly susceptible to disruption by external factors (e.g., modulation of protease activity, transcriptional inhibition). Once this equilibrium is disturbed, even minor perturbations can trigger toxin release.

#### 2.3.3 Potential intervention strategies

A successful curing agent must be capable of “de-addiction,” enabling bacteria to survive following plasmid loss.

##### 2.3.3.1 Direct detoxification

This involves developing small molecules that can directly bind to the active site of the toxin protein, acting as exogenous “antidotes” to neutralize toxin activity. For instance, specific small molecules could disrupt the stability of the toxin-antitoxin complex or directly inhibit the toxic effects of the toxin (Równicki et al., 2020).

##### 2.3.3.2 Blocking antitoxin degradation

Under normal circumstances, once a plasmid is lost, the labile antitoxin is rapidly degraded by host proteases (e.g., Lon or Clp), leading to toxin activation and bacterial cell death. If the activity of these proteases can be inhibited, the degradation of the antitoxin can be prevented, allowing it to persist and continuously neutralize the toxin. Consequently, even after plasmid loss, bacteria would not die from the “withdrawal effect,” thereby permitting the survival of plasmid-free, susceptible strains.

### 2.4 Conjugal transfer

Conjugal transfer is the primary pathway for the horizontal dissemination of plasmids among bacteria and a key mechanism for the rapid spread of drug resistance. This process is highly dependent on plasmid-encoded type IV secretion systems (T4SSs) and relaxosome complexes, providing well-defined molecular targets for the development of inhibitors that block transmission.

#### 2.4.1 Mating pair formation and the type IV secretion system

Conjugal transfer initiates with the physical contact between a donor and a recipient cell. This process is mediated by the plasmid-encoded T4SS. The T4SS is a complex transmembrane protein assembly that is responsible not only for assembling the connecting sex pilus but also for forming the channel for DNA translocation. The assembly and function of the T4SS rely on the coordinated action of various VirB/VirD4 homologous proteins, particularly the energy-providing Tra ATPases. Disruption of the assembly or ATP hydrolysis activity of these core proteins can directly block mating pair formation (MPF) (Cabezón et al., 2015).

#### 2.4.2 DNA processing and relaxase

Concurrently with the establishment of the T4SS connection, plasmid DNA must undergo specific processing to enable its transfer. Relaxase is the key enzyme in this process. With the assistance of accessory proteins, it specifically recognizes and cleaves the phosphodiester bond at the plasmid transfer origin (oriT). This cleavage generates a single-stranded DNA molecule (T-strand). Subsequently, the relaxase covalently attaches to the 5′ end of the T-strand, guiding it through the T4SS channel into the recipient cell. Within the recipient, the single-stranded DNA recircularizes, and a complementary strand is synthesized, thereby completing the transfer (Cabezón et al., 2015).

#### 2.4.3 Target relevance

Although blocking conjugation does not eliminate plasmids within the host, it effectively curbs their dissemination within the bacterial community, constituting a crucial component of the “disarmament” strategy (Cabezón et al., 2017). The selection of targets for blocking conjugation is primarily based on their non-essentiality for host physiological functions.

##### 2.4.3.1 Non-essentiality of the channel

The T4SS is a molecular machine specifically evolved for the secretion of DNA or effector proteins. Unlike fundamental transport channels for nutrient uptake, inhibiting the T4SS typically does not affect the basic metabolism and growth of bacteria. This provides a wide therapeutic window for T4SS inhibitors.

##### 2.4.3.2 Uniqueness of the enzyme

The relaxase is the sole rate-limiting enzyme that initiates the DNA transfer reaction. Within the host genome, homologous enzymes with functions completely overlapping those of plasmid relaxases are generally absent. Therefore, targeting the active site of the relaxase enables precise inhibition of plasmid transfer without off-target effects.

#### 2.4.4 Potential intervention strategies

The “disarming” strategy targeting conjugation transfer focuses on interrupting the transmission pathway:

*Channel blockade*: This approach involves designing molecules to interfere with the protein–protein interactions of T4SS core components (e.g., VirB8) or to inhibit the ATPase (Tra ATPase) that energizes the transport process, thereby physically blocking the DNA transfer channel. This strategy has been demonstrated to possess significant potential in suppressing the dissemination of drug resistance (Álvarez-Rodríguez et al., 2020).

*Enzymatic inhibition*: The key tyrosine residue within the active site of the relaxase is central to its catalytic DNA cleavage activity. Intervention strategies include: (1) Competitive inhibition: Utilizing small molecules (e.g., bisphosphonates) that mimic the DNA phosphate backbone to competitively occupy the enzyme’s active site pocket; (2) Covalent/allosteric inhibition: Employing molecules with specific reactive groups (e.g., fatty acids containing unsaturated bonds or natural active compounds) to inactivate the enzyme through direct modification or allosteric regulation. These approaches would effectively block the horizontal transfer of resistance plasmids from donor to recipient bacteria.

## 3 Mechanisms of plasmid curing by natural products

As discussed in Section 2, multiple specific intervention strategies theoretically exist that target key processes in plasmid maintenance and transfer. The “multi-target” mode of action exhibited by natural products confers unique characteristics compared to synthetic intercalating dyes or phenothiazine drugs (Ferrari et al., 2004; Motohashi et al., 1992). With their distinctive chemical diversity and complex stereochemistry, natural products provide a rich material basis for validating these “theoretical strategies.” Unlike the often singular mode of action of synthetic drugs, natural phytochemicals frequently demonstrate curing activities that align closely with the aforementioned theoretical strategies (e.g., topological interference, enzyme inhibition, channel blockade) through synergistic, multi-target mechanisms. This section will elaborate on how various classes of natural products specifically implement these intervention mechanisms.

### 3.1 Pleiotropic mechanisms of natural products

While this review focuses on the plasmid-curing mechanisms of natural products, it is essential to acknowledge their pleiotropic nature. Natural products often act as multi-target agents. For instance, berberine is a well-documented inhibitor of the bacterial cell division protein FtsZ and various RND-family efflux pumps (e.g., MexAB-OprM) (Boberek et al., 2010; Duda-Madej et al., 2025). Curcumin exhibits primary antimicrobial and synergistic activities by directly downregulating and inhibiting efflux pumps (such as AcrAB-TolC and MexAB-OprM) and disrupting bacterial membranes (Dai et al., 2022; Rahbar Takrami et al., 2019). Terpenes like thymol and carvacrol strongly perturb lipid bilayer membranes and act as competitive inhibitors of the NorA efflux pump (Dos Santos Barbosa et al., 2021; Kachur and Suntres, 2020). Furthermore, ascorbic acid (vitamin C) exerts profound synergistic and antibiofilm effects through the generation of ROS, quorum sensing disruption, and induction of severe oxidative stress (Shivaprasad et al., 2021; Wen et al., 2025). Therefore, the observed plasmid loss in natural product assays is often a component of a broader, multi-faceted antibacterial response rather than an isolated event.

### 3.2 Inhibition of plasmid replication via DNA interaction, damage, or enzyme inhibition

The mechanisms described in this section directly correspond to the strategies of “topological interference” and “competitive inhibition” proposed in Section 2.1.4. By physically intercalating into or competitively binding to DNA, natural products disrupt the topological environment or enzymatic conditions required for Rep protein recognition of the origin of replication (ori). Based on this principle, many bioactive molecules derived from plants exhibit exceptional DNA affinity or enzyme inhibitory activity, which constitutes the core molecular basis for their ability to eliminate drug-resistance plasmids. In-depth structure–activity relationship (SAR) analyses have revealed that the specific chemical scaffolds of these molecules determine their modes of interaction with plasmid DNA.

#### 3.2.1 DNA intercalation and binding mechanisms

Certain phytochemicals with planar aromatic structures (e.g., alkaloids, anthraquinones) can function as DNA intercalating agents. From a SAR perspective, this rigid planar structure allows the molecules to insert between DNA base pairs via *π*-π stacking interactions. Furthermore, many alkaloids carry a positive charge (e.g., quaternary ammonium groups), enabling electrostatic attraction with the negatively charged phosphate groups on the DNA backbone, which further stabilizes the binding. This physical intercalation leads to DNA helix unwinding, elongation, and conformational rigidity, thereby interfering with the normal binding and progression of replication machinery such as DNA polymerases and helicases. Additionally, some compounds (e.g., curcumin, polyphenols) preferentially bind to the DNA minor groove. Although this non-covalent binding does not disrupt base pairing, it induces subtle conformational distortions in the DNA, which are sufficient to hinder the recognition of the plasmid replication origin by Rep proteins. This mechanism can specifically block plasmid replication without affecting the bacterial chromosome.

#### 3.2.2 DNA structural damage and breakage

In addition to physical binding, certain natural products (e.g., ascorbic acid, tannic acid) can induce oxidative stress under specific conditions (such as in the presence of transition metal ions), generating ROS. These ROS can attack the DNA backbone, leading to the breakage of plasmid DNA. This damage converts the supercoiled plasmid (the replication-competent form) into open-circular or linear forms (replication-incompetent forms), directly resulting in the loss of the plasmid during bacterial cell division.

#### 3.2.3 Inhibition of key replicative enzymes

DNA gyrase is an essential enzyme in bacterial DNA replication, responsible for introducing negative supercoils to relieve topological tension ahead of the replication fork. Natural products such as coumarins and flavonoids have been demonstrated to be potent DNA gyrase inhibitors. They competitively bind to the ATP-binding pocket of the enzyme (e.g., the GyrB subunit) or stabilize the enzyme-DNA complex (e.g., the GyrA subunit), thereby blocking the topological adjustments necessary for plasmid replication and ultimately leading to plasmid curing.

The specific mechanisms of action of various natural products are as follows:

*Alkaloids*: In this context, berberine, derived from Coptis chinensis, is the most extensively studied and profound alkaloid. Research indicates that berberine exhibits significant antimicrobial activity, which is closely associated with its mechanism of interfering with DNA synthesis and function. It can effectively eliminate resistance plasmids in bacteria (Čerňáková and Košťálová, 2002). Structurally, the planar configuration of the isoquinoline ring in berberine provides the spatial geometry required for intercalation between DNA base pairs, while the positive charge on its quaternary ammonium nitrogen atom enhances electrostatic interactions with the negatively charged DNA backbone, rendering it a potent DNA intercalator. Furthermore, related studies have confirmed that berberine can reverse the drug resistance of multidrug-resistant bacteria by inhibiting efflux pumps and through direct plasmid elimination (Spengler et al., 2006). Similarly, total alkaloids from *Sophora flavescens* (primarily containing matrine, oxymatrine, etc.) have also been demonstrated to effectively reverse drug resistance in ESBL-producing *Escherichia coli*. The mechanism involves the inhibition of resistance gene expression and plasmid elimination (Zhou et al., 2013).

*Quinones*: Plumbagin, derived from *Plumbago zeylanica*, is a selective curing agent. Studies have demonstrated its particular efficacy against the F′ plasmid in *Escherichia coli*, and it can cure plasmids such as TP181 and R162 with an elimination rate of up to 100%(Patwardhan et al., 2018). The mechanism involves the specific inhibition of DNA synthesis in plasmid-containing cells, exploiting the metabolic burden imposed by the plasmid. Furthermore, lawsone has been purified and identified as the active component responsible for the curing activity in *Plumbago zeylanica* extracts (Patwardhan et al., 2018).

*Anthraquinones*: Emodin, which is widely present in *Rheum palmatum*, possesses a characteristic tricyclic planar skeleton. SAR analysis indicates that this rigid planar framework serves as the structural basis for its function as a DNA intercalator, allowing the molecule to insert tightly between DNA base pairs via *π*–π stacking interactions. The emodin molecule can intercalate between DNA base pairs, not only inhibiting the activity of nucleic acid polymerases but also generating ROS through redox cycling, thereby inducing plasmid DNA strand breaks. This effectively blocks plasmid replication and promotes its elimination (Khan and Hadi, 1998).

*Flavonoids*: Certain planar flavonoids have been demonstrated to interact with DNA via a “mixed-mode” mechanism, which concurrently involves intercalation and groove binding. SAR studies indicate that the planarity of the flavonoid scaffold is crucial. Specifically, the presence of the C2 = C3 double bond in the C-ring maintains the molecular coplanarity, enabling intercalation between base pairs. Concurrently, the hydroxyl substitution pattern on the B-ring influences its capacity for hydrogen bonding within the DNA minor groove. For instance, quercetin and kaempferol, which are widely present in various medicinal plants, have been shown to bind with high affinity to calf thymus DNA (Oyedemi et al., 2019). This binding alters the DNA conformation and impedes the progression of the replicase complex.

*Saponins*: Recent studies have demonstrated that madecassic acid, a triterpenoid saponin derived from *Centella asiatica*, exhibits significant DNA-binding capacity. Ultraviolet absorption spectroscopy analysis revealed that madecassic acid induces a red shift and hypochromic effect in DNA, which are characteristic features of DNA intercalative binding. This interaction results in the relaxation of bacterial DNA supercoiling, converting it into open-circular or linear DNA, thereby blocking normal plasmid replication (Wei et al., 2023).

*Polyphenols/Curcuminoids*: Curcumin, the active constituent of *Curcuma longa*, has been demonstrated to specifically bind to the minor groove of DNA and induce plasmid DNA damage via photodynamic effects or oxidative stress. It can cause a conformational change in plasmids from the supercoiled form to the nicked open-circular form, thereby effectively inhibiting replication (Priyadarsini, 2014).

*Coumarins*: Coumarins and their derivatives are well-known inhibitors of bacterial DNA gyrase. Specifically, they competitively bind to the ATP-binding pocket of the GyrB subunit, inhibiting its ATPase activity and thereby preventing the introduction of DNA supercoiling. A natural coumarin isolated from Mesua ferrea has demonstrated potent anti-plasmid activity against *Salmonella Typhi* (Maxwell, 1993).

*Tannins*: Hydrolyzable tannins, such as tannic acid and ellagic acid, exhibit plasmid elimination activity through multiple mechanisms. They can bind to DNA, inducing conformational changes. More importantly, in the presence of certain metal ions (e.g., Cu^2+^), they generate reactive oxygen species via the Fenton reaction, leading to single-strand scission of plasmid DNA (Khan et al., 2000).

*Diterpenes*: 8-Epidiosbulbin E acetate (EEA) is a norditerpenoid compound isolated from the tubers of *Dioscorea bulbifera*. Studies have shown that EEA can specifically interfere with plasmid replication, thereby effectively eliminating RP4, pUB110, and R136 plasmids in clinical isolates of *Escherichia coli* and *Pseudomonas aeruginosa*. Notably, while exhibiting significant plasmid elimination activity, EEA did not show obvious cytotoxicity, indicating that its mechanism of action is highly selective for plasmid replication (Shriram et al., 2008).

*Ascorbic acid (Vitamin C)*: Although primarily utilized as a vitamin, ascorbic acid has been demonstrated under specific conditions to induce single-strand breaks in plasmid DNA by generating reactive oxygen species, converting supercoiled plasmid DNA into non-replicable open circular forms. This mechanism effectively eliminates penicillinase-encoding plasmids in *Staphylococcus aureus* (Amábile-Cuevas et al., 1991).

### 3.3 Disruption of membrane potential and bioenergetics: increasing the metabolic burden of plasmids

This mechanism represents an indirect implementation of the strategy described in Section 2.2.3, “Inhibiting the Motility Engine.” By disrupting the transmembrane proton motive force (PMF), natural products cut off the ATP supply required for ParA ATPase activity and T4SS assembly, thereby paralyzing the plasmid’s partitioning and maintenance systems. This is because the replication, partitioning, and maintenance of bacterial plasmids are highly energy-consuming processes that critically depend on the host cell’s ATP supply and a stable transmembrane PMF. Certain lipophilic phytochemical constituents can target the bacterial cell membrane, interfering with the energy metabolism system via non-lethal membrane damage. From a SAR perspective, the membrane-targeting ability of these compounds is typically determined by their lipophilicity and amphiphilic characteristics, which enable them to embed into the lipid bilayer. Molecules containing weakly acidic groups, such as phenolic hydroxyl groups, are particularly noteworthy as they can act as protonophores, shuttling protons across the membrane and directly leading to the dissipation of the proton gradient. When bacteria are in an energy-depleted state of stress, they initiate resource reallocation mechanisms to prioritize survival. This includes the elimination of “non-essential and metabolically burdensome” genetic elements, such as drug resistance plasmids, through mechanisms like post-segregational loss.

#### 3.3.1 Disruption of membrane integrity and ion leakage

Lipophilic terpenoid and phenolic compounds can insert into the lipid bilayer of the bacterial cell membrane, increasing membrane fluidity and permeability. This leads to the non-specific leakage of crucial ions such as potassium ions and protons, resulting in the dissipation of the membrane potential.

#### 3.3.2 ATP depletion and plasmid instability

The PMF serves as the primary driver for bacterial ATP synthesis. Its dissipation inhibits the function of ATP synthase, leading to the depletion of intracellular ATP pools. Since the normal function of plasmid replication initiation proteins (Rep) and partition proteins (ParA/ParB) is dependent on ATP, energy deficiency directly blocks plasmid replication and accurate segregation. This ultimately promotes the elimination of the plasmid from the bacterial population.

The following are representative natural products under this mechanism:

*Essential oils*: Thymol and carvacrol possess protonophore activity, capable of dissipating pH gradients and membrane potential. This activity is attributed to their unique chemical structure: the hydrophobic isopropyl group enables insertion into the hydrophobic core of the bacterial cell membrane, while the phenolic hydroxyl group serves as a proton exchange site, transporting protons from the periplasmic space into the cytoplasm, thereby disrupting the PMF. This perturbation of bioenergetics leads to the elimination of plasmids carrying tetracycline resistance genes due to insufficient replication energy (Sousa Silveira et al., 2020). Furthermore, coriander oil, by compromising membrane integrity, successfully induced the loss of R plasmids in *Pseudomonas* spp. and *Escherichia coli* at sub-inhibitory concentrations (Schelz et al., 2006). Additionally, lavender oil (*Lavandula angustifolia*) has been demonstrated to effectively reverse plasmid-mediated drug resistance by disrupting cell membrane integrity and inhibiting quorum sensing (Yap et al., 2014).

*Phloroglucinols*: Rottlerin, isolated from the medicinal plant *mallotus philippensis*, is a potent bacterial membrane uncoupler. By disrupting oxidative phosphorylation, it severs the supply of ATP. In an *Escherichia coli* model, this energy blockade directly induced the destabilization and elimination of the pKM101 and TP114 plasmids without significantly inhibiting bacterial growth (Oyedemi et al., 2016).

*Polyphenols*: Epigallocatechin gallate (EGCG) is the primary active component in green tea. Studies have shown that EGCG can bind to peptidoglycan on the bacterial cell membrane, compromising cell wall integrity and interfering with membrane-associated energy metabolism processes. This leads to the destabilization and loss of resistance plasmids in methicillin-resistant *Staphylococcus aureus* (MRSA) (Zhao et al., 2001).

*Triterpenoid saponins*: Certain triterpenoid saponins (e.g., asiatic acid) possess an amphiphilic structure, consisting of a hydrophobic triterpenoid backbone (aglycone) and a hydrophilic sugar chain. This structure confers surfactant-like properties, enabling them to insert into bacterial cell membranes, reduce membrane surface tension, and alter membrane permeability. This membrane disruption leads to the leakage of critical ions such as potassium and a decrease in transmembrane potential. At sub-inhibitory concentrations, this alteration in energy status forces bacteria to cease plasmid replication and maintenance to sustain basic survival, thereby resulting in plasmid curing (Wei et al., 2023).

### 3.4 Inhibition of conjugation: disarming transmission

As established in Section 2.4, disrupting the T4SS is critical for halting the horizontal spread of multidrug resistance. The natural products detailed in this section provide concrete material evidence for these anti-conjugation strategies. Rather than directly eliminating plasmids within the donor host, these compounds specifically disarm the transfer machinery. At the molecular level, they achieve this by covalently modifying and locking the relaxase, allosterically closing the T4SS channel, or interfering with interbacterial QS communication. Notably, many of these natural inhibitors possess highly reactive pharmacophores—such as unsaturated double bonds or Michael acceptors—capable of selective binding to the active sites of key conjugation proteins.

*Inhibition of MPF*: Bacterial conjugation requires the donor cell to assemble sex pili via the T4SS and establish a physical connection with the recipient cell. Natural inhibitors can block the assembly or ATPase activity of core T4SS proteins (e.g., VirB/VirD4 homologs), thereby physically preventing DNA transfer.

*Inhibition of relaxase*: The initial step of conjugative transfer involves the cleavage of DNA strands at the plasmid transfer origin (oriT) by relaxase. Inhibition of relaxase expression or activity directly immobilizes the plasmid, preventing its entry into the recipient bacterium in a single-stranded form.

*Quorum quenching*: Plasmid transfer is typically regulated by cell density. Natural products can render bacteria “blind” by mimicking or degrading signaling molecules (such as AHLs or AI-2), thereby downregulating the expression of conjugation-related genes (tra genes).

The specific mechanisms of action of various natural products are as follows:

*Unsaturated fatty acids*: Specific fatty acids such as 2-hexadecynoic acid and oleic acid are potent inhibitors of conjugation. SAR analysis indicates that the inhibitory activity of fatty acids is strictly dependent on the position and stereochemistry of the double bond. The “kink” structure introduced into the hydrocarbon chain by a cis-double bond may disrupt the fluidity of membrane microdomains responsible for T4SS assembly. Furthermore, specific chain lengths and unsaturation (e.g., an alkyne bond) may exhibit high spatial complementarity with the substrate-binding pocket of the TrwD ATPase, leading to specific inhibition. Mechanistic studies have shown that they specifically bind to and inhibit TrwD ATPase, a traffic ATPase within the T4SS responsible for energizing DNA transport. This inhibition occurs at nanomolar concentrations and effectively blocks the conjugative transfer of IncF and IncW plasmids in *Escherichia coli*, with a low propensity for the development of drug resistance (Getino et al., 2015).

*Phenylpropanoids*: 1’-Acetoxychavicol acetate (ACA) is a phytochemical derived from *Alpinia galanga*. The *α*, *β*-unsaturated carbonyl moiety present in the ACA molecule acts as an active michael acceptor, readily undergoing Michael addition reactions with key nucleophilic residues (such as the thiol group of cysteine) in conjugation regulatory proteins. This results in the irreversible inhibition of protein function through covalent modification. ACA exhibits dual activity: it can both eliminate plasmids and potently inhibit conjugation. Studies have shown that ACA significantly downregulates the gene expression of plasmid-encoded transfer proteins, thereby inhibiting the conjugative transfer of vancomycin-resistant plasmids in *Enterococcus faecalis* with an inhibition rate of up to 92% (Li et al., 2025).

*Flavonoids*: A groundbreaking study in 2025 confirmed that baicalin can inhibit the conjugative transfer of the multidrug-resistant RP4 plasmid. The mechanism involves its specific binding to the LsrB protein (the AI-2 transport receptor). By competitively occupying the receptor, baicalin blocks AI-2 signal communication between bacteria, leading to the comprehensive downregulation of genes regulating conjugative transfer (Patwardhan et al., 2018).

*Capsaicinoids*: Bioactive constituents such as capsaicin from chili peppers and gingerol from ginger have been demonstrated to inhibit the conjugative transfer of R plasmids (pKM101, TP114) in *Escherichia coli*. These compounds primarily interfere with the gene expression or function of the relaxase, thereby preventing the conversion of plasmid DNA from the supercoiled to the single-stranded transfer form and consequently blocking the transmission pathway (Oyedemi et al., 2019).

*Polyketides and marine*: Beyond traditional terrestrial plants, recent advances have identified potent conjugation inhibitors from marine and fungal environments. For instance, tanzawaic acids A and B, polyketides derived from the marine fungus Penicillium sp., exhibit highly specific inhibitory activity against IncFII and IncW conjugative plasmids without affecting bacterial growth. Strikingly, recent *in vivo* validation (2021) utilizing 2-hexadecynoic acid (a mechanistically related fatty acid conjugation inhibitor) demonstrated a remarkable 50-fold reduction in plasmid conjugation frequency within a complex mouse gut model (Palencia-Gándara et al., 2021). Furthermore, marine-derived compounds are expanding the “disarmament” arsenal; specific fatty acids (myristic and oleic acids) isolated from the marine sponge *Mycale contarenii* have been recently (2023) shown to profoundly repress virulence and biofilm-related genes (fnbA/B and icaADBC) in MRSA without bactericidal selective pressure (Khan et al., 2023). These non-plant sources represent a vast, untapped reservoir for novel curing and anti-conjugation scaffolds.

*Natural complexes*: Recent studies have demonstrated that certain natural extracts or their nano-formulations can effectively interfere with bacterial conjugation. For instance, nanostructured lipid carriers loaded with sage and olibanum essential oils have been shown to significantly reduce plasmid conjugation frequency. This effect is potentially mediated by influencing membrane stability or the assembly of conjugative pili, offering a novel perspective for alternative antibiotic therapies (Guidotti-Takeuchi et al., 2023).

### 3.5 Interference with plasmid partitioning: disrupting the final defense of genetic stability

This section directly corresponds to the strategy of “Disrupting the Positioning Complex” outlined in Section 2.2.3. Natural products interfere with cytoskeletal proteins (e.g., FtsZ), thereby disrupting the spatial anchoring required by the Par system, which leads to the abandonment of plasmids during cell division. The biological basis for this is that low-copy-number plasmids rely on a precise partitioning system (Par system) to ensure their accurate segregation into daughter cells during bacterial division. The canonical Par system typically consists of an ATPase (ParA), a CTPase/DNA-binding protein (ParB), and a cis-acting site (parS). Natural products cause plasmid partitioning failure by inhibiting the polymerization, assembly, or nucleotide (ATP/CTP) hydrolysis activity of these key proteins.

*Interference with cytoskeletal assembly*: The Z-ring, formed by the bacterial cell division protein FtsZ, is not only responsible for cell division but also serves as a critical anchor for the localization of plasmid partitioning systems. Inhibition of FtsZ polymerization or its GTPase activity impedes Z-ring formation, thereby disrupting the spatial coordination required for plasmid partitioning and leading to increased plasmid instability in daughter cells.

*Blockade of partitioning protein function*: The formation and operation of the plasmid partition complex rely on the energy cycle and conformational changes of Par proteins (e.g., the ParA ATPase and ParB CTPase). Interfering with the nucleotide binding, hydrolysis, or conformational stability of these proteins directly blocks the precise segregation of plasmids into daughter cells, leading to their loss during cell division.

The specific mechanisms of action of various natural products are as follows:

*Polyphenols*: Curcumin, extracted from *Curcuma longa*, potently inhibits the assembly of the bacterial cell division protein FtsZ and reduces its GTPase activity. This treatment induces bacterial filamentation and disrupts Z-ring formation, thereby indirectly blocking the accurate spatial segregation of plasmids into daughter cells (Rai et al., 2008).

*Alkaloids*: Berberine, beyond its previously detailed DNA intercalation, recent biochemical experiments reveal its role as a novel FtsZ inhibitor. Berberine binds to the FtsZ protein with high affinity, impeding Z-ring contraction. This interference disrupts the spatial coordination essential for plasmid partitioning, leading to severe plasmid instability (Boberek et al., 2010).

*Phenylpropanoids*: Cinnamaldehyde, the primary active constituent in cinnamon bark, is a potent FtsZ inhibitor. Its highly reactive terminal aldehyde group interacts with nucleophilic residues within the FtsZ active site. This effectively inhibits GTPase activity and polymerization dynamics, disrupting the proper localization of the partition complex at the division plane (Domadia et al., 2007).

*Quinones*: Plumbagin exerts a profound elimination effect on F-like plasmids by disrupting both replication and maintenance. Mechanistic studies suggest that plumbagin alter the functional conformation of Par proteins or depletes their necessary ATP supply. This directly prevents the formation of the partition complex, leading to plasmid abandonment during cell division (Chiang et al., 1996).

*Fungal and marine metabolites*: Recent screens have identified potent non-plant partitioning inhibitors. The fungal metabolite 4-hydroxy-sterigmatocystin (4HS) directly targets the ParB protein, specifically inhibiting its parS-dependent CTPase activity and disrupting phase separation (Zhao H. et al., 2024). Similarly, the marine product psammaplysin F disrupts chromosomal partitioning against MRSA (Ramsey et al., 2013), highlighting the untapped potential of diverse ecological niches.

## 4 Screening strategies and methodological workflows: from phenotype assays to high-throughput platforms

Establishing a rigorous and scientifically sound evaluation pipeline is paramount for the discovery of novel plasmid-curing agents. Historically, the field has struggled to distinguish true “plasmid curing” (the targeted elimination of a plasmid) from “selective killing” (general bactericidal effects against plasmid-bearing cells). To address this critical bottleneck and meet the demands of modern pharmacology, evaluation methodologies have transitioned from classical phenotypic verification to multidimensional workflows that integrate high-throughput screening, precise molecular quantification, and functional transmission blockade (Table 1).

**Table 1:** Summary of natural products with plasmid curing and anti-conjugation activities.

Chemical class	Plant source	Active compound	Molecular target/Mechanism	Cured plasmid/Effect	References
Alkaloids	Coptis chinensis	Berberine	DNA intercalation (Planar Cationic); FtsZ inhibition	R-plasmids; Inhibition of cell division	Boberek et al. (2010)
	*Sophora flavescens*	Matrine/Oxymatrine	Down-regulation of resistance genes (ESBLs/qnrS); Efflux pump inhibition	Reversal of antibiotic resistance	Hu et al. (2025)
Anthraquinones	*Rheum palmatum*	Emodin	DNA intercalation (Tricyclic skeleton); ROS generation	Elimination of R-plasmids; DNA strand breaks	Chen (1985) and Li et al. (2010)
Quinones	*Plumbago zeylanica*	Plumbagin	Interference with Par system stability; Replication inhibition	F′ plasmid, R136, pUPI281	Patwardhan et al. (2018)
Flavonoids	Scutellaria baicalensis	Baicalin	Quorum Sensing inhibition (Targets LsrB receptor)	RP4 plasmid conjugation inhibition	Li et al., 2025
	Various plants	Quercetin/Kaempferol	DNA interaction (Intercalation & Groove binding)	General resistance plasmids	Cushnie and Lamb (2005)
Phenylpropanoids	*Alpinia galanga*	1’-Acetoxychavicol acetate (ACA)	Covalent modification of Tra proteins (Michael acceptor)	Inhibition of conjugation (pRE25)	Latha et al. (2009)
	*Cinnamomum* spp.	Cinnamaldehyde	FtsZ polymerization inhibition (Reactive aldehyde group)	Disruption of plasmid partitioning	Chai et al. (2022)
Curcuminoids	*Curcuma longa*	Curcumin	FtsZ assembly inhibition; DNA damage (ROS/Nicking)	Filamentation; Plasmid loss	Rai et al. (2008) and Tyagi et al. (2015)
Coumarins	Mesua ferrea	Coumarins	DNA gyrase inhibition (GyrB subunit)	Salmonella R-plasmids	Maxwell (1993) and Oyedemi et al. (2019)
Diterpenes	*Dioscorea bulbifera*	8-Epidiosbulbin E acetate (EEA)	Plasmid replication machinery interference	RP4, pUB110, R136	Shriram et al. (2008)
Essential Oils	*Thymus vulgaris*	Thymol/Carvacrol	Protonophore; Membrane potential (PMF) dissipation	Tetracycline resistance plasmids	Bonetti et al. (2022)
	*Lavandula angustifolia*	Lavender oil	Membrane integrity disruption; QS inhibition	Reversal of antibiotic resistance	Fernández Iriarte et al. (2020)
Fatty Acids	General	2-Hexadecynoic acid/Oleic acid	TrwD ATPase inhibition; Membrane fluidity (Cis-double bond)	IncF, IncW plasmids (Conjugation inhibition)	Fernandez-Lopez et al. (2005)
Capsaicinoids	*Capsicum annuum*	Capsaicin/Gingerol	Relaxase inhibition or expression downregulation	Inhibition of R-plasmid (pKM101) transfer	Oyedemi et al. (2019)
Saponins	*Centella asiatica*	Madecassic acid	Membrane permeability (Pore formation); DNA interaction	General plasmid curing	Wei et al. (2023)
Polyketides	Marine fungi	Tanzawaic acids	Conjugation complex assembly inhibition	IncFII, IncW plasmids	Getino et al. (2016)
Tannins	Various plants	Tannic acid/Ellagic acid	DNA strand scission (ROS via Fenton reaction)	Plasmid curing	Bhat and Hadi (1994)
Polyphenols	*Camellia sinensis*	EGCG	Membrane bioenergetics disruption (ATPase inhibition)	Membrane bioenergetics disruption (ATPase inhibition) MRSA plasmid loss/Resistance reversal	Dadi et al. (2009) and Zhao et al. (2001)
Others	Synthetic/Natural	Ascorbic acid (Vitamin C)	DNA strand scission (ROS generation)	*S. aureus* plasmids	Amábile-Cuevas et al. (1991)
	Natural complexes	Sage/Olibanum Nano-oils	Membrane stability/Pili assembly interference	Conjugation inhibition	Guidotti-Takeuchi et al. (2023)

### 4.1 The gold standard for phenotypic verification: replica plating

Despite being time-consuming, the phenotype-based replica plating method remains the “gold standard” for confirming the loss of drug resistance to date. Schelz et al. validated and applied this standardized procedure when investigating the plasmid-curing activity of plant essential oils (natural products).

#### 4.1.1 Determination of the subinhibitory concentration (SIC)

The fundamental prerequisite for validating any curing agent is ensuring that the observed plasmid loss is not an artifact of host cell death. Therefore, researchers must meticulously determine the Minimum Inhibitory Concentration (MIC) and the Non-Inhibitory Concentration (NIC). Subsequent curing assays strictly utilize subinhibitory concentrations (typically 0.25–0.5 × MIC) to completely abrogate the confounding interference of bactericidal or bacteriostatic effects (Lambert and Pearson, 2000).

#### 4.1.2 Replica plating and differential screening

Although labor-intensive, the phenotype-based replica plating technique remains the definitive “gold standard” for confirming the phenotypic loss of antibiotic resistance. Following a 24- to 48-h incubation with the curing agent, the bacterial culture is diluted and plated onto non-selective agar to obtain single colonies. These colonies are then precisely transferred—using sterile velvet pads or automated multi-pin replicators—onto selective plates containing the plasmid-targeted antibiotic. The curing efficiency is quantitatively expressed as the percentage of colonies that proliferate on the master plate but fail to survive under selective pressure (Park et al., 2024).

### 4.2 High-throughput and single-cell analysis: fluorescence-based screening

Because classical replica plating lacks the scalability required for screening extensive natural product libraries, the field has increasingly adopted fluorescence reporter systems to achieve rapid, HTS.

#### 4.2.1 Construction of fluorescently reporter systems

This approach involves cloning genes encoding fluorescent proteins (such as GFP or RFP) under the control of constitutive promoters directly into the target multidrug-resistant plasmid. These engineered reporter plasmids exhibit exceptional *in vitro* and *in vivo* stability, enabling the real-time, non-destructive tracking of plasmid maintenance and segregation (Rodriguez et al., 2017).

#### 4.2.2 Microplate-based dual-readout HTS

Automated primary screening of natural products is typically executed in 96- or 384-well microplates using a multi-mode plate reader. By continuously monitoring the ratio of fluorescence intensity to optical density (OD600), researchers can successfully decouple true plasmid elimination (indicated by a disproportionate loss of fluorescence) from generalized growth inhibition (proportional decline in both parameters) (Zulauf and Kirby, 2020).

#### 4.2.3 Single-cell flow cytometry analysis

Flow cytometry allows for the direct quantification of fluorescent plasmid loss at single-cell resolution. Beyond simply measuring fluorescence, this technique simultaneously captures structural parameters—such as forward scatter and side scatter—to monitor drug-induced morphological aberrations, such as bacterial filamentation resulting from FtsZ inhibition. This step is crucial for ruling out indirect plasmid loss caused by compromised cell division (Wickens et al., 2000).

### 4.3 Genotypic validation and precise quantification: qPCR workflows

#### 4.3.1 Absolute quantification of plasmid copy number (PCN)

While traditional agarose gel electrophoresis offers only semi-quantitative insights, real-time quantitative PCR (qPCR) has emerged as the definitive method for capturing the dynamic fluctuations of PCN. By calculating the ratio between the amplification threshold cycle (Ct) of a single-copy plasmid target (e.g., repA or bla) and a strictly conserved chromosomal housekeeping gene (e.g., dxs or 16S rRNA), researchers can precisely map the dose-dependent reduction of PCN prior to complete plasmid eviction (Lee et al., 2006).

#### 4.3.2 Definitive PCR validation of resistance clearance

To definitively confirm that the “cured” isolates have been entirely stripped of their resistance determinants, conventional multiplex PCR is employed. This final validation step ensures the complete genotypic eradication of highly transmissible resistance genes (such as mcr-1 or blaCTX-M) from the bacterial population (Jousset et al., 2019).

### 4.4 Assessment of transmission blockade: conjugation inhibition assays

For natural products (e.g., unsaturated fatty acids) that target the T4SS or relaxase rather than replication, their efficacy must be evaluated through standardized horizontal gene transfer blockade assays.

#### 4.4.1 Establishment of the donor-recipient model

The assay necessitates a rigorously defined donor strain (harboring the conjugative resistance plasmid) and a distinct recipient strain. To unequivocally differentiate transconjugants from the parental strains, the recipient is typically engineered or selected to carry a unique chromosomal resistance marker (such as sodium azide or rifampicin resistance) (Getino et al., 2015).

#### 4.4.2 Determination of conjugation frequency (liquid/filter mating)

Donor and recipient populations are co-cultured at optimized ratios under sub-inhibitory concentrations of the candidate drug, utilizing either liquid suspension or solid filter mating protocols. Following the mating period, the mixture is plated onto dual-antibiotic selective agar—targeting both the plasmid-encoded trait and the recipient’s chromosomal marker—to specifically isolate successful transconjugants (Simonsen et al., 1990).

### 4.5 Calculation of inhibition efficacy

The absolute conjugation frequency is mathematically defined as the number of transconjugants per recipient or donor cell. The anti-conjugation potency of the natural product is then rigorously quantified by comparing the calculated conjugation frequency of the treatment group against a vehicle-treated control, thereby yielding the final transmission inhibition rate (Fernandez-Lopez et al., 2005).

## 5 Synergistic effects and clinical relevance

The ultimate clinical goal of plasmid elimination is not merely to clear the episomal DNA, but to definitively reverse antibiotic resistance. Natural curing agents can serve as potent “antibiotic adjuvants” or synergists. When combined with existing antibiotics, they can restore the bactericidal efficacy of these antibiotics against drug-resistant bacteria, providing a novel strategy for clinical treatment.

### 5.1 Restoring antibiotic susceptibility: the cornerstone of combination therapy

Studies have consistently demonstrated that natural resistance breakers can reduce the MIC of conventional antibiotics by several-fold, even rendering drug-resistant bacteria susceptible again (Skulj et al., 2008). This “antibiotic adjuvant” strategy provides a novel approach for clinical combination therapy. For instance, in MRSA, the combination of flavonoids (such as rutin, morin, and quercetin) with antibiotics can produce significant additive or synergistic effects, substantially lowering the required antibiotic concentration (Amin et al., 2015). This synergistic effect not only enhances therapeutic efficacy but also reduces the required antibiotic dosage, thereby mitigating side effects and the further development of drug resistance.

### 5.2 Preliminary evidence of *in vivo* efficacy

Although most studies remain at the ex vivo stage, some *in vivo* experiments have demonstrated the potential of natural plasmid eliminators. For instance, in a mouse thigh infection model with multidrug-resistant *Acinetobacter baumannii*, the combination therapy of berberine and sulbactam exhibited stronger antibacterial efficacy compared to monotherapy, significantly reducing the bacterial load in the tissue (Li et al., 2021). These studies indicate that natural eliminators can retain activity within complex in vivo environments and hold promise as effective adjunctive agents in combating drug-resistant bacterial infections.

### 5.3 Synergistic multi-target effects: a comprehensive strategy against drug-resistant bacteria

Unlike synthetic agents (e.g., SDS or phenothiazines) (Motohashi et al., 1992), natural products typically exhibit multi-target effects, combating drug-resistant bacteria through multiple mechanisms. In addition to curing plasmids, they often possess the ability to inhibit bacterial efflux pumps, disrupt biofilms, or modulate host immune responses. For instance, natural products acting as efflux pump inhibitors (EPIs) can block the extrusion of antibiotics from bacterial cells. A recent review highlighted that plant-derived EPIs can serve as novel potentiators to control drug-resistant pathogens by restoring intracellular drug concentrations (Zhang et al., 2023). This synergistic, multi-mechanistic action confers a distinct advantage to natural products in combating complex, drug-resistant bacterial infections.

### 5.4 Recent advances in nano-delivery: overcoming bioavailability bottlenecks

A major hurdle in translating natural plasmid-curing agents into clinical practice is their poor aqueous solubility and rapid *in vivo* degradation. Recently, advanced nano-delivery systems—such as liposomes and lipid-chitosan nanocapsules—have emerged as significant breakthroughs to encapsulate these bioactives.

For example, co-encapsulating berberine and curcumin into combinatorial liposomes decreased the required MIC of berberine by 87% and successfully reduced intracellular MRSA infections by 77% (Bhatia et al., 2021). Similarly, a 2024 study demonstrated that loading novel coumarins into nanocapsules astonishingly suppressed the MIC by 65-fold compared to the free compound, causing severe bacterial cell wall disruption (Radwan et al., 2024). Moving forward, actively fusing these highly efficient drug-delivery platforms with specific *in vivo* plasmid curing assays will be a crucial next step.

### 5.5 Challenges and prospects in clinical translation

Despite the promising prospects and recent advances in nano-delivery, translating these findings into clinical therapies faces several challenges, including drug bioavailability, toxicity, and regulatory hurdles. Future development efforts need to focus on discovering novel scaffolds and optimizing existing compounds to overcome these bottlenecks in clinical translation (Chawla et al., 2022). With in-depth research into the mechanisms of action of natural products and advancements in pharmaceutical technologies, natural plasmid-curing agents are expected to become an important component of future anti-infective therapies.

## 6 Challenges and perspectives

The clinical translation of natural product-based plasmid curing agents is currently hindered by several critical bottlenecks. To move beyond preclinical models, future research must overcome challenges ranging from conceptual conflations in the literature to poor pharmacokinetic profiles in *in vivo* applications.

### 6.1 The critical gap: conflating resistance reversal with true *in vivo* curing

A critical analysis of recent literature reveals a profound gap in true in vivo plasmid curing. Much of the current research frequently conflates genuine plasmid elimination with mere resistance reversal or conjugation inhibition. For instance, while natural products like rottlerin successfully reduce the conjugation frequency of clinically relevant blaNDM-1-carrying plasmids (e.g., pCPE16_3 in *K. pneumoniae*), they strictly prevent horizontal transfer rather than eradicating the resident plasmids from the donor host (Alav et al., 2024). Furthermore, quantitative data demonstrating the complete elimination of critical MDR plasmids by pure natural products in animal models is remarkably scarce. Most phenotypic curing assays remain confined to *in vitro* settings using reference laboratory plasmids rather than clinically isolated superbugs. Moving forward, the field must strictly differentiate between conjugation blockade and true plasmid loss, and urgently transition from in vitro screening to rigorous in vivo quantification using murine or Galleria mellonella infection models.

### 6.2 Overcoming the narrow therapeutic window

A major hurdle is the conflict between the curing concentration and host toxicity. The effective SIC for plasmid curing often overlaps with cytotoxic concentrations for mammalian cells (e.g., non-specific DNA intercalators causing genotoxicity). Medicinal chemistry efforts must focus on improving the “selectivity index” by shifting away from non-specific DNA interactions toward highly specific PPI inhibitors targeting critical plasmid nodes like ParA and Rep.

### 6.3 *In vivo* efficacy and nanotech-based delivery

Addressing the aforementioned in vivo gap, some of the most robust demonstrations of true plasmid curing currently rely on nanomedicine rather than pure phytochemicals. For example, sub-lethal levels of platinum nanoparticles successfully cured ESBL-harboring plasmids in a zebrafish infection model, achieving a 2.4 log reduction in bacterial burden and restoring carbapenem susceptibility in vivo (Bharathan et al., 2019). To overcome the poor aqueous solubility, rapid gastrointestinal degradation, and low bioavailability inherent to many natural products, future studies must aggressively explore advanced nano-drug delivery systems. Platforms such as lipid nanocarriers and polymeric nanoparticles can provide sustained, targeted delivery, widening the therapeutic window and minimizing systemic toxicity.

### 6.4 Integration of cutting-edge technologies

#### 6.4.1 CRISPR-Cas9 synergies

The primary translational bottleneck for CRISPR-based plasmid curing is cellular delivery efficiency. Recent advances highlight that combining Cas effectors with membrane-permeabilizing nanomaterials can overcome these barriers. In this context, natural curing agents can act as synergistic “priming perturbants” by disrupting bacterial biofilms or efflux pumps, thereby maximizing the delivery and editing efficiency of CRISPR systems against multidrug-resistant plasmids.

#### 6.4.2 AI and machine learning

By training models on high-throughput screening data, AI can now predict in silico interactions across massive chemical libraries (>100,000 compounds). Landmark successes (such as the discovery of halicin and abaucin) illustrate how AI-guided screening can unlock the untapped potential of vast natural product databases for plasmid curing.

### 6.5 Overcoming “addiction systems” (TA systems)

While inhibitors of replication and partitioning are well-documented, a critical conceptual void exists in targeting plasmid TA systems. Current research often mistakenly focuses on activating toxins to kill cells, which contradicts the core “curing” philosophy. True plasmid curing requires neutralizing the Toxin or artificially stabilizing the Antitoxin to prevent post-segregational killing when the plasmid is lost. In the future, leveraging recent structural predictions and synthetic biology approaches to guide the screening of natural small molecules that specifically neutralize toxins will significantly enhance the curing efficiency of highly stable, low-copy-number plasmids.

## 7 Conclusion

Confronted with the “silent pandemic” of AMR, traditional “bactericidal” strategies have exposed their inherent limitations, often accelerating the evolutionary trajectory of resistance. By recognizing drug-resistance plasmids as the primary engines of horizontal gene transfer, the natural product-based “plasmid curing” strategy represents a fundamental paradigm shift in anti-infective therapy—from violent eradication to ecologically intelligent “genetic disarmament.” By selectively targeting the genetic vectors of resistance, this strategy not only holds the promise of restoring the efficacy of legacy antibiotics but also curtails the dissemination of resistance at its evolutionary source.

However, as critically analyzed in this review, the field must rapidly evolve beyond phenomenological observations and the simple cataloging of bioactive extracts. The successful translation of these natural agents depends entirely on overcoming the “therapeutic window paradox.” Future research must shift focus from non-specific DNA intercalators, which carry inherent host genotoxicity, toward the rational design of highly specific PPI inhibitors targeting critical plasmid nodes (e.g., Rep, ParA/ParB, and relaxases). Furthermore, it is imperative that the field strictly adopts standardized, multidimensional methodological workflows to definitively differentiate true *in vivo* plasmid curing from mere selective bactericidal effects.

Looking ahead, bridging the substantial gap between *in vitro* promise and clinical application requires the aggressive integration of multidisciplinary technologies. The deployment of AI and machine learning for high-throughput structural predictions, combined with advanced nanomedicine to resolve the inherent pharmacokinetic limitations of natural products, will dramatically accelerate drug discovery. Furthermore, exploring synergistic applications with CRISPR-Cas9 systems opens entirely new therapeutic avenues. By transforming these critical insights into actionable research frameworks, natural plasmid-curing agents are poised to become indispensable “antibiotic companions,” offering a sustainable and crucial line of defense for safeguarding global biosecurity in the post-antibiotic era.
